# Impacts of Neighborhood Characteristics and Surgical Treatment Disparities on Overall Mortality in Stage I Renal Cell Carcinoma Patients

**DOI:** 10.3390/ijerph19042050

**Published:** 2022-02-12

**Authors:** Alejandro Cruz, Faith Dickerson, Kathryn R. Pulling, Kyle Garcia, Francine C. Gachupin, Chiu-Hsieh Hsu, Juan Chipollini, Benjamin R. Lee, Ken Batai

**Affiliations:** 1 Department of Urology, University of Arizona, Tucson, AZ 85724, USA; acruz700@surgery.arizona.edu (A.C.); fdickerson@email.arizona.edu (F.D.); kpulling@email.arizona.edu (K.R.P.); kylegarcia1@arizona.edu (K.G.); jchipollini@arizona.edu (J.C.); brlee@arizona.edu (B.R.L.); 2 Department of Family and Community Medicine, University of Arizona, Tucson, AZ 85711, USA; fcgachupin@arizona.edu; 3 Department of Epidemiology and Biostatistics, University of Arizona, Tucson, AZ 85724, USA; pchhsu@arizona.edu

**Keywords:** kidney cancer, cancer health disparities, surgical disparities, neighborhood socioeconomic status, geospatial

## Abstract

Racial/ethnic minority groups in the United States have high renal cell carcinoma (RCC) mortality rates. This study assessed surgical treatment disparities across racial/ethnic groups and impacts of neighborhood socioeconomic characteristics on surgical treatments and overall mortality. Stage I RCC patients diagnosed between 2004 and 2016 from National Cancer Database were included (n = 238,141). We assessed differences in associations between race/ethnicity and treatment patterns using logistic regression and between race/ethnicity and overall mortality using Cox regression with and without neighborhood characteristics in the regression models. When compared to non-Hispanic Whites (NHWs), American Indians/Alaska Natives and non-Hispanic Blacks (NHBs) were more likely not to receive surgical care and all racial/ethnic minority groups had significantly increased odds of undergoing radical rather than partial nephrectomy, even after adjusting for neighborhood characteristics. Including surgical treatment and neighborhood factors in the models slightly attenuated the association, but NHBs had a significantly increased risk of overall mortality. NHBs who underwent radical nephrectomy had an increased risk of mortality (HR 1.15, 95% CI: 1.08–1.23), but not for NHBs who underwent partial nephrectomy (HR 0.92, 95% CI: 0.84–1.02). Neighborhood factors were associated with surgical treatment patterns and overall mortality in both NHBs and NHWs. Neighborhood socioeconomic factors may only partly explain RCC disparities.

## 1. Introduction

Racial/ethnic minority groups in the United States (U.S.) have a disproportionate kidney cancer burden with high incidence and mortality rates [1,2,3]. Many small renal masses, as well as early-stage kidney cancer, can be resected through partial nephrectomy, a less invasive surgical treatment than radical nephrectomy [4]. The rate of partial nephrectomy for the treatment of early-stage kidney cancer has increased over time, while the rate of radical nephrectomy has gradually declined [5,6,7]. Previous studies have reported surgical treatment disparities across racial/ethnic minority groups for renal cell carcinoma (RCC), the most common type of kidney cancer, as well as for other cancer types [5,8,9,10,11,12]. Disparities in RCC surgical treatment may contribute to higher RCC mortality in racial/ethnic minority groups when compared to non-Hispanic Whites (NHWs), but the relationship between surgical treatment disparities and disparities in RCC mortality is not well understood [10,13]. Biological factors may contribute to disparities in kidney cancer mortality [14,15,16]; however, socioeconomic characteristics are also considered to be major contributing factors for RCC surgical treatment disparities [7,17]. Structural inequality reflected in neighborhood-level socioeconomic factors and residential segregation has been linked to cancer mortality rates [18,19,20,21,22]. However, the way socioeconomic factors affect RCC surgical treatment and mortality has not been fully investigated.

In this study, we assessed disparities that might exist in surgical treatments across racial/ethnic groups and the way neighborhood socioeconomic characteristics may account for the surgical disparities among the TNM (tumor, node, metastasis) Stage I renal cell carcinoma (RCC) patients. Then, we assessed the impact of neighborhood socioeconomic characteristics and surgical treatments on overall mortality.

## 2. Materials and Methods

### 2.1. Patients Data

Clinicopathologic and demographic information of RCC patients who were diagnosed between 2004 and 2016 was obtained from National Cancer Database (NCDB). The NCDB, jointly sponsored by the American College of Surgeons and the American Cancer Society, is a clinical oncology database containing data collected from more than 1500 Commission on Cancer (CoC)-accredited facilities in the U.S. and Puerto Rico. Since 1996, all CoC-accredited programs have been required to report cancers diagnosed and treated at their facilities to the NCDB. The CoC-accredited hospitals generally are higher volume centers than non-accredited centers, and cases from accredited hospitals account for 70% of cancers diagnosed annually in the U.S. The NCDB has standardized data pertaining to patient demographics, tumor characteristics, surgery performed, primary treatment, and mortality. This study focused on patients with RCC; kidney cancer patients with pathological type other than RCC were excluded. We also focused on the Stage I patients based on American Joint Committee on Cancer (AJCC) staging system using pathologic stage group, since surgical treatment recommendations differ based on the stage (tumor size) [4]. We excluded patients that were reported as being unstaged or missing a stage of tumor. De-identified data were obtained from the NCDB, so the Institutional Review Board approval was not necessary for this project.

Only cases with known race/ethnicity were included in this study. Heterogeneity among Hispanic Americans (HAs) was assessed based on their origin (Mexico, Puerto Rico, Cuba, South or Central America, and Dominican Republic). Neighborhood-level socioeconomic variables used were educational attainment (proportion of adults who did not graduate from high school), and median household income based on zip code from the 2012 American Community Survey data (2008–2012). Patients’ level and nature of access to healthcare were assessed using insurance type, facility type, the 2013 U.S. Department of Agriculture Economic Research Service Rural-Urban Continuum Codes (RUCC), and “great circle” distance (distance in miles between zip code of patient’s residence and the hospital that reported the case). This study assessed three outcome variables: (1) not receiving surgical treatment versus undergoing surgical treatment (local ablation or nephrectomy; (2) undergoing more invasive surgical treatment (radical nephrectomy) versus partial nephrectomy; (3) overall mortality.

### 2.2. Statistical Analysis

Chi-squared tests were used to compare the characteristics of RCC patients across racial/ethnic minority groups and HA subgroups. To assess if neighborhood characteristics account for racial/ethnic disparities in RCC surgical treatment (no treatment vs. local ablation/nephrectomy as well as radical vs. partial nephrectomy), logistic regression analysis was performed. First, patient’s demographic and healthcare information (such as age category, gender, RCC histologic subtype, facility type, insurance type, Charlson/Deyo Score, and year of diagnosis) were included in the regression model (Model 1). In the final model (Model 2), we additionally included RUCC (metro counties, urban counties, or rural counties), great circle distance (every 10 miles increment), high school education (21% or more, 13–20.9%, 7–12.9%, or less than 7%), and/or median household income (less than $38,000, $38,000–$47,999, $48,000–$62,999, or $63,000 or more). All the variables included in the final regression model were significantly associated with surgical treatment (at least one category in the variables with *p* < 0.05 in adjusted models).

Similarly, a Cox regression analysis was performed to assess if neighborhood characteristics and surgical treatment account for racial/ethnic disparities in overall mortality. To further assess effect of surgical disparities on association between race/ethnicity and overall mortality, analysis was performed stratifying samples based on surgical treatment. Sub-analysis was performed for non-Hispanic Blacks (NHBs) and NHWs to assess the relationship between neighborhood socioeconomic factors and both surgical treatment patterns and overall mortality in each racial group separately. The final models in sub-analysis included variables that were significantly associated with treatment patterns or overall mortality in adjusted models. Statistical analysis was performed with IBM SPSS Statistics Version 27 (IBM, Armonk, NY, USA).

## 3. Results

### 3.1. Characteristics of Patients

A total of 238,141 patients were included in the analysis (Table 1). In all racial/ethnic groups, there were more males than females (>50% in all racial/ethnic group). Most patients presented with Grade 1 and 2 RCC (about 75% in all racial/ethnic groups). NHWs, American Indians/Alaska Natives (AI/ANs), Asian Americans, and HAs presented commonly with clear cell RCC histologic subtype (>70%). NHBs presented commonly with either clear cell or papillary RCC (43.3 and 42.0%). Asian Americans were more likely to have private insurance (48.9%) when compared to the other racial/ethnic groups. Other racial/ethnic groups were more likely to have public insurance. (>46%). A high proportion of NHWs and AI/ANs were treated in a comprehensive community cancer program whereas patients from all other racial/ethnic groups were commonly treated at academic/research program. Many patients from all racial/ethnic groups, except for AI/ANs, resided in metropolitan areas. NHWs and Asian American were more likely to live in neighborhoods with a median income greater than $63,000 (34.2 and 55.7%). A high percentage of AI/ANs and NHBs lived in neighborhood with a median income lower than $38,000. NHBs and HAs were more likely to live in neighborhoods with a high rate of no high school degree (≥21%) when compared to all other racial/ethnic groups (33% for both groups).

HAs were then broken down into subgroups (Table S1). Cubans were found to have the highest median age at 64, while Mexican/Chicanos and South/Central Americans were found to have the lowest median age at 58. Clear cell RCC was the most common histological subtype. Dominicans had a higher percentage of papillary RCC (29.9%) than other HA subgroups. Many HAs subgroups had public insurance and lived in neighborhood with low median income and high school education graduation rates. Most subgroups used academic research programs for their treatment facility. However, 51.3% of Cubans were found to use integrated network cancer programs as their main source of treatment facility. Most patients from all subgroups lived in metropolitan areas, rather than rural or urban areas.

### 3.2. No Surgical Treatment vs. Local Ablation/Surgery

When compared to all other racial/ethnic minority groups, NHBs were more likely to receive no treatment (10.1%) (Table 2). NHWs had the highest rate of local ablation (9.4%). Nephrectomy was common among all racial/ethnic groups (>80%). When compared to NHWs, AI/ANs and NHBs were more likely not to receive surgical care, even adjusting for neighborhood socioeconomic factors (OR 1.85, 95% Cl: 1.28–2.70 and OR 1.32 95% Cl: 1.20–1.45 respectively). This association was slightly attenuated after including healthcare access and neighborhood factors. We further analyzed variation in types of treatment among HA subgroups. Dominicans presented with the highest percentage for receiving no treatment (14.5%). Cubans had the highest rate in undergoing local ablation (9.3%). A large majority of HA patients in all HA subgroups underwent nephrectomy (>78% in all Hispanic subgroups). No associations were found to be significant when comparing odds of no surgical treatment in each individual HA subgroup to NHW.

### 3.3. Radical vs. Partial Nephrectomy

When compared to NHWs, NHBs, AI/Ans, and HAs were significantly more likely to undergo radical nephrectomy as opposed to partial nephrectomy (Table 3). The associations were slightly attenuated when we included healthcare access and neighborhood factors for AI/ANs, NHBs, and HAs, but remained significant. The association became significant for Asian Americans after including healthcare access and neighborhood factors. Stratifying by neighborhood characteristics yielded similar results, but the association was not significant for AI/ANs living in neighborhoods with higher income and high school graduation rates (Table S2). Mexican/Chicanos had the highest proportion of patients undergoing radical nephrectomy (52.1%) and significantly increased odds of undergoing radical nephrectomy even after adjusting for level and nature of healthcare access and neighborhood characteristics (OR 1.29, 95% CI: 1.14–1.47). South/Central Americans were less likely to undergo radical nephrectomy (OR 0.81, 95% CI: 0.66–0.98).

### 3.4. Overall Mortality

Patients who did not receive surgical treatment had a higher risk of mortality (HR 5.76, 95% CI: 5.62–5.91), and this also held true in our adjusted model (HR 3.02, 95% CI: 2.80–3.26). Undergoing radical nephrectomy was associated with higher overall mortality in both unadjusted and adjusted models (unadjusted HR 1.81 95% CI: 1.77–1.86 and adjusted HR 1.52, 95% CI: 1.47–1.58). NHBs had an elevated risk of overall mortality, while Asian Americans and HAs had reduced risk (Table S3). Including surgical treatment, health access and neighborhood factors in the model did not affect the association between race/ethnicity and overall mortality. In HA subgroup analysis, Mexican/Chicanos, Puerto Ricans, and South or Central Americans had a lower risk of overall mortality. When including surgical treatments, healthcare access and neighborhood factors, the associations for Puerto Ricans remained significant.

Analysis was performed upon stratifying samples based on surgical treatment to assess the effect of surgical treatment disparities on the association between race/ethnicity and overall mortality (Table 4; Table S4). Among patients who underwent surgical treatment, NHBs had an increased risk of mortality when compared to NHWs (HR 1.11, 95% CI: 1.06–1.17), but there was no increased risk of mortality in NHBs who did not receive surgical treatment. In contrast, HAs had a decreased risk of mortality among patients who had surgical treatment (HR 0.87, 95% CI: 0.83–0.91). Among patients who underwent radical nephrectomy, NHBs had an increased risk of mortality (HR 1.15, 95% CI: 1.08–1.23), but interestingly there was no significant difference in mortality between NHBs and NHWs who underwent partial nephrectomy (HR 0.93, 95% CI: 0.84–1.02). Asian Americans and Puerto Ricans who underwent partial nephrectomy had significantly reduced risk of mortality. HA had lower mortality when compared to NHWs in patients who underwent radical nephrectomy (HR 0.87, 95% CI: 0.82–0.93) and partial nephrectomy (HR 0.81, 95% CI: 0.73–0.89).

### 3.5. Sub-Analysis in NHBs and NHWs

Finally, we performed sub-analyses in NHBs and NHWs to further assess impacts of neighborhood socioeconomic factors on surgical treatment and overall mortality in each group. Living in higher income neighborhoods was associated with reduced odds of no treatment in both NHBs and NHWs (Table S5). Living in neighborhoods with low percentages of individuals without high school education and higher income were significantly associated with reduced odds of radical nephrectomy in NHWs, but not in NHBs. Living in higher median income neighborhoods was significantly associated with reduced overall mortality in both groups, but neighborhood-level high school graduation rates were associated with overall mortality only in NHWs only (Table S6).

## 4. Discussion

Our study of Stage I RCC patients revealed that NHBs and AI/ANs were less likely to undergo treatment for RCC, and racial/ethnic minority patients who did undergo treatment were more likely to undergo more invasive surgical treatment, including radical nephrectomy, even when controlling for healthcare access factors and neighborhood-level socioeconomic characteristics. HAs, particularly Mexican/Chicanos, were also more likely to undergo radical nephrectomy. NHBs who underwent radical nephrectomy had an increased risk of mortality, but there were no disparities among patients who underwent partial nephrectomy. Neighborhood socioeconomic factors were significantly associated with surgical treatment patterns and overall mortality in both NHBs and NHWs. Adding neighborhood socioeconomic factors in regression models did not eliminate the disparities in surgical treatment and overall mortality between NHBs and NHWs.

Previous studies have demonstrated that racial/ethnic minority cancer patients are less likely to receive surgical treatment for cancer when compared to NHW patients [9,10,12,23]. This study also found that racial/ethnic minority patients were less likely to undergo surgical treatment for RCC. Racial/ethnic minority patients may have chosen active surveillance over surgical treatment for their initial management [24], and effects of socioeconomic and healthcare access factors on selecting active surveillance across racial/ethnic groups need further investigation. AI/ANs living in neighborhoods with higher income and high school graduation rates did not have significantly elevated odds of undergoing radical nephrectomy, suggesting that improving social determinants of health may lead to equitable care for AI/ANs. However, our study demonstrated that racial/ethnic minority groups had higher odds of undergoing radical nephrectomy rather than partial nephrectomy even when factoring in patient’s demographics, comorbidities, level and nature of healthcare access, and neighborhood-level socioeconomic factors. Previous literature supports our findings regarding NHBs and HAs having higher odds of undergoing radical nephrectomies [5,8,25]. Another study reported that NHB females, in particular, were more likely to undergo radical nephrectomy rather than partial nephrectomy [26]. There are many factors that may influence whether or not a patient undergoes treatment and which surgical treatment they undergo, including prognosis, age, comorbidities, insurance status, distance to a treatment facility, and socioeconomic background. Including these factors in the regression models did not eliminate the disparities in this study. Interactions of these factors may have contributed to surgical treatment disparities. For example, lower-income minority patients may have insurance that covers providers who do not specialize in performing partial nephrectomies [27]. However, interactions of various factors have not been investigated. It is also likely that additional factors, such as baseline renal function, tumor size, and complexity contribute to this [28]. Our analysis, further adjusting for tumor size (≤4 cm vs. 4–7 cm), yielded similar findings. Furthermore, “race” is a social construct, and “race” encompasses various unmeasured factors, such as psychosocial and cultural variables, trust issues, and discrepancies in patient–physician communication, which may play a role in the ultimate determination of whether patients undergo surgical treatment or not, whether they select active surveillance, and which surgical treatment they undergo [29,30]. Future studies are necessary to understand how these factors may influence treatment decisions.

As with many other studies, this study found that NHBs had higher overall mortality when compared to NHWs [31,32,33], and these previous studies suggested the socioeconomic difference between two groups as a major reason for the disparities. In their study of Detroit Surveillance, Epidemiology and End Results (SEER) data, Schwartz and colleagues showed that including socioeconomic factors in the model eliminated the survival disparities [33]. In another study of a single-payer healthcare system, race was not associated with survival [34]. In our study, neighborhood-level socioeconomic factors predicted overall mortality in both groups, and contrary to the previous findings, including socioeconomic factors did not eliminate the disparities. We further demonstrated that NHBs who underwent radical nephrectomy had an increased risk of overall mortality when compared to their NHW counterparts, but there was no difference in mortality between NHWs and NHBs who underwent partial nephrectomy. Chronic kidney disease is more common in NHB RCC patients [34,35], and patients who undergo nephrectomy often develop chronic kidney disease [36,37]. Equitable surgical care for NHB patients with minimally invasive surgical treatment to preserve kidney function may reduce these disparities.

A previous study reported similar rates of surgical treatment for HAs and NHWs [38]. Unlike this study in a single-payer healthcare system, our sub-analysis focusing on HA subgroups showed that Mexicans/Chicanos had higher odds of undergoing radical nephrectomy and South or Central Americans had lower odds of undergoing radical nephrectomy. Higher kidney cancer mortality rates in Mexicans when compared to non-Mexican Hispanic groups or NHWs have been reported [2,39]. However, in NCDB, HAs tend to have a lower risk of overall mortality. NCDB is a hospital-based registry, and racial/ethnic minority patients are underrepresented, while patients from academic and comprehensive cancer programs are overrepresented [40,41]. Future studies are necessary to understand the effects of variation in sociocultural factors and healthcare access across HA subgroups on surgical treatment and mortality [42].

There are some limitations of the current study. First, NCDB is a hospital-based registry, and the findings cannot be projected to non-CoC hospitals [41]. Our study findings need to be validated with population-based data, such as the SEER database or state cancer registry data. We previously reported inconsistent findings for mortality between NCDB and Arizona Cancer Registry data in HAs [39]. Arizona Cancer Registry data showed an increased risk of mortality in Mexican Americans, which is consistent with other studies [2,43], but lack of association for Mexican Americans and reduced risk for HA as a group in NCDB. Regional differences or incomplete follow-up in the administrative data may have contributed to this difference, and further investigation is necessary for HAs [44]. Second, detailed clinical characteristics and comorbidity information that are used for clinical decision-making are not available in the NCDB [28]. Specifically, lack of preoperative renal function, which could impact decision-making between partial versus radical nephrectomy, was not assessed due to limitations in the NCDB. Including these factors in regression may eliminate the observed disparities. Moreover, we limited our analysis to Stage I cases with known race/ethnicity, and excluding unstaged cases and cases with missing stages and race/ethnicity information may have biased the samples. Racial/ethnic minority patients have higher rates of unstaged cancer than NHW patients [45,46]. Finally, this study assessed the impact of neighborhood-level socioeconomic status on RCC disparities. The NCDB does not have data on individual-level socioeconomic factors, and individual-level differences in socioeconomic factors may better explain the RCC disparities. Kidney cancer mortality rates in the U.S. are increasing over 15 years for all racial/ethnic groups [47]. Further studies are required to understand the causes of kidney cancer treatment disparities and their relation to mortality.

## 5. Conclusions

Racial/ethnic minority patients were more likely not to receive surgical treatment. When they do, they are likely to have less optimal surgical treatment (radical rather than partial nephrectomy). Neighborhood-level socioeconomic factors may only partly explain the disparities in surgical treatment and overall mortality. Surgical treatment disparities may account for high RCC mortality in NHBs. Having equitable kidney cancer care for NHBs and other racial/ethnic minority groups and being aware of factors affecting their medical care will lead to better considerations in treatments provided.

## Figures and Tables

**Table 1:** Stage I RCC patient characteristics across racial/ethnic groups.

Characteristics of Patients, n (%)	NHW (n = 176,478)	AI/AN (n = 1017)	NHB (n = 28,647)	Asian American (n = 3722)	HA (n = 28,277)	*p*
Age, median (IQR)	63 (54–72)	57 (48–67)	60 (52–68)	61 (51–70)	60 (50–69)	<0.001
Gender, n (%)						<0.001
Male	107,671 (61.0)	545 (53.6)	16,493 (57.6)	2361 (63.4)	16,390 (58.0)	
Female	68,807 (39.0)	472 (46.4)	12,154 (42.4)	1361 (36.6)	11,887 (42.0)	
Grade, n (%)						<0.001
1 & 2	101,982 (76.4)	655 (79.8)	14,911 (74.0)	2118 (74.9)	17,161 (78.9)	
3 & 4	31,461 (23.6)	166 (20.2)	5245 (26.0)	711 (25.1)	4581 (21.1)	
Histologic Subtype, n (%)						<0.001
Clear Cell	89,997 (72.3)	655 (87.2)	8486 (43.3)	2208 (78.6)	14,470 (75.0)	
Papillary	21,577 (17.3)	48 (6.4)	8244 (42.0)	323 (11.5)	2719 (14.1)	
Chromophobe	8816 (7.1)	23 (3.1)	1579 (8.1)	189 (6.7)	1451 (7.5)	
Other	4103 (3.3)	25 (3.3)	1302 (6.6)	90 (3.2)	659 (3.4)	
Insurance Type, n (%)						<0.001
Private	80,948 (45.9)	324 (31.9)	10,540 (36.8)	1819 (48.9)	12,691 (44.9)	
Public	88,715 (50.3)	627 (61.7)	16,362 (57.1)	1736 (46.6)	13,178 (46.6)	
Not insured	3611 (2.0)	33 (3.2)	1178 (4.1)	106 (2.8)	1635 (5.8)	
Unknown	3204 (1.8)	33 (3.2)	567 (2.0)	61 (1.6)	773 (2.7)	
Facility Type						<0.001
Community Cancer Program	12,305 (7.3)	106 (11.3)	1426 (5.2)	278 (8.0)	1757 (6.7)	
Comprehensive Community Cancer Program	70,523 (42.0)	434 (46.4)	8302 (30.5)	1031 (29.8)	10,385 (39.9)	
Academic/Research Program	66,999 (39.9)	327 (35.0)	13,527 (49.8)	1884 (54.5)	10,998 (42.2)	
Integrated Network Cancer Program	18,226 (10.8)	68 (7.3)	3924 (14.4)	263 (7.6)	2898 (11.1)	
County-level Residence Pattern						<0.001
Metropolitan	138,967 (81.2)	498 (49.6)	25,451 (90.8)	3530 (97.3)	24,204 (87.6)	
Urban	28,494 (16.6)	401 (39.9)	2297 (8.2)	96 (2.6)	3062 (11.1)	
Rural	3695 (2.2)	105 (10.5)	287 (1.0)	3 (0.1)	378 (1.4)	
Median Income Quartiles						<0.001
<$38,000	24,134 (13.8)	422 (41.9)	11,650 (40.9)	230 (6.2)	6435 (22.9)	
$38,000–$47,999	41,645 (23.8)	268 (26.6)	6383 (22.4)	461 (12.5)	6677 (23.8)	
$48,000–$62,999	49,420 (28.2)	203 (20.1)	5697 (20.0)	948 (25.6)	7713 (27.5)	
$63,000+	59,912 (34.2)	115 (11.4)	4728 (16.6)	2063 (55.7)	7262 (25.9)	
% No High School Degree						<0.001
≥21%	22,348 (12.8)	310 (30.7)	8407 (33.0)	769 (20.8)	9118 (33.0)	
13.0–20.9%	44,718 (25.5)	327 (32.4)	9960 (35.0)	746 (20.1)	6973 (24.8)	
7.0–12.9%	62,357 (35.6)	270 (26.7)	6419 (22.5)	1121 (30.3)	7277 (25.9)	
<7.0%	45,765 (26.1)	103 (10.2)	2686 (9.4)	1067 (28.8)	4730 (16.8)	

Note: Abbreviation: NHW, non-Hispanic White; AI/AN, American Indian/Alaska Native; NHB, non-Hispanic Black; HA, Hispanic American, IQR, interquartile range. *p*-values from Chi-square test.

**Table 2:** Logistic regression assessing association with no surgical treatment vs. local ablation/nephrectomy.

Race/Ethnicity	No Treatment, n (%)	Local Ablation, n (%)	Nephrectomy, n (%)	Model 1		Model 2	
				OR (95% CI)	*p*	OR (95% CI)	*p*
NHW	12,957 (7.4)	16,506 (9.4)	146,692 (83.3)	Reference		Reference	
AI/AN	76 (7.5)	89 (8.8)	850 (83.7)	1.94 (1.34–2.80)	<0.001	1.85 (1.28–2.70)	0.001
NHB	2881 (10.1)	2264 (7.9)	23,422 (82.0)	1.44 (1.32–1.57)	<0.001	1.32 (1.20–1.45)	<0.001
Asian American	238 (6.4)	231 (6.2)	3247 (87.4)	0.92 (0.71–1.18)	0.51	0.92 (0.71–1.18)	0.51
HA	1965 (7.0)	2186 (7.8)	24,030 (85.3)	1.06 (0.96–1.17)	0.26	1.02 (0.92–1.13)	0.72
NHW				Reference		Reference	
Mexican/Chicano	148 (7.6)	125 (6.4)	1679 (86.0)	1.03 (0.73–1.44)	0.89	0.97 (0.68–1.37)	0.85
Puerto Rican	42 (7.9)	39 (7.3)	454 (84.9)	0.64 (0.30–1.37)	0.25	0.63 (0.29–1.34)	0.23
Cuban	50 (11.6)	40 (9.3)	340 (79.1)	1.17 (0.60–2.30)	0.65	1.13 (0.58–2.22)	0.72
South or Central American	62 (7.3)	39 (4.6)	746 (88.1)	0.86 (0.48–1.53)	0.60	0.87 (0.49–1.55)	0.63
Dominican	24 (14.5)	12 (7.2)	130 (78.3)	0.32 (0.44–2.30)	0.26	0.31 (0.04–2.27)	0.25

Model 1 adjusted for age category, gender, RCC histologic subtype, facility type, insurance type, Charlson/Deyo Score, and year of diagnosis. Model 2 adjusted for age category, gender, RCC histologic subtype, facility type, insurance type, Charlson/Deyo Score, year of diagnosis, urban/rural residence, and neighborhood characteristics (median income).

**Table 3:** Logistic regression assessing undergoing radical nephrectomy vs. partial nephrectomy.

Race/Ethnicity	Radical, n (%)	Partial, n (%)	Model 1		Model 2	
			OR (95% CI)	*p*	OR (95% CI)	*p*
NHW	59,797 (45.9)	70,507 (54.1)	Reference		Reference	
AI/AN	389 (51.8)	362 (48.2)	1.31 (1.09–1.56)	0.003	1.27 (1.06–1.52)	0.01
NHB	10,128 (49.6)	10,271 (50.4)	1.46 (1.40–1.52)	<0.001	1.38 (1.33–1.44)	<0.001
Asian American	1309 (44.1)	1659 (55.9)	1.12 (1.03–1.23)	0.01	1.16 (1.06–1.27)	0.01
HA	10,578 (50.0)	10,578 (50.0)	1.16 (1.11–1.20)	<0.001	1.12 (1.05–1.26)	0.002
NHW			Reference		Reference	
Mexican/Chicano	776 (52.1)	713 (47.9)	1.36 (1.20–1.54)	<0.001	1.29 (1.14–1.47)	<0.001
Puerto Rican	154 (37.9)	252 (62.1)	0.96 (0.75–1.22)	0.75	0.93 (0.73–1.19)	0.59
Cuban	145 (47.2)	162 (52.8)	0.93 (0.71–1.22)	0.59	0.88 (0.67–1.16)	0.37
South or Central American	265 (38.9)	416 (61.1)	0.84 (0.69–1.02)	0.07	0.81 (0.66–0.98)	0.03
Dominican	42 (36.2)	74 (63.8)	0.78 (0.49–1.24)	0.29	0.73 (0.45–1.18)	0.20

Model 1 adjusted for age category, gender, RCC histologic subtype, facility type, insurance type, Charlson/Deyo Score, and year of diagnosis. Model 2 adjusted for age category, gender, RCC histologic subtype, facility type, insurance type, Charlson/Deyo Score, year of diagnosis, urban/rural residence, great circle distance, and neighborhood characteristics (median income and high school education).

**Table 4:** Effect of nephrectomy type on association between race/ethnicity and overall survival.

Race/Ethnicity	Radical Nephrectomy		Partial Nephrectomy		
	HR (95% CI)	*p*	HR (95% CI)	*p*	*p*_-Interaction_
					<0.001
NHW	Reference		Reference		
AI/AN	0.90 (0.65–1.26)	0.55	0.93 (0.57–1.52)	0.76	
NHB	1.15 (1.08–1.23)	<0.001	0.92 (0.84–1.02)	0.12	
Asian American	0.74 (0.61–0.91)	0.003	0.58 (0.44–0.78)	<0.001	
HA	0.87 (0.82–0.93)	<0.001	0.81 (0.73–0.89)	<0.001	
					0.04
Non-Hispanic Whites	Reference		Reference		
Mexican/Chicano	0.88 (0.70–1.10)	0.26	0.97 (0.69–1.36)	0.86	
Puerto Rican	1.00 (0.60–1.67)	0.99	0.18 (0.36–0.57)	0.003	
Cuban	0.89 (0.57–1.41)	0.63	0.60 (0.30–1.21)	0.15	
South or Central American	0.77 (0.48–1.22)	0.26	0.72 (0.44–1.20)	0.21	
Dominican	0.81(0.37–1.82)	0.62	0.37 (0.05–2.62)	0.32	

Regression model includes age category, gender, RCC histologic subtype, grade (1 and 2 vs. 3 and 4), facility type, insurance type, great circle distance, neighborhood characteristics (median income and % high school graduation), Charlson/Deyo Score, and year of diagnosis.

## Data Availability

The data used in this project is available from the NCDB (https://www.facs.org/Quality-Programs/Cancer/NCDB, accessed on 25 October 2017).

## Supplementary Materials

The following are available online at https://www.mdpi.com/article/10.3390/ijerph19042050/s1, Table S1: Stage I RCC patient characteristics across Hispanic subgroups, Table S2 Logistic regression assessing undergoing radical nephrectomy vs. partial nephrectomy stratified by neighborhood characteristics, Table S3: Cox Regression analysis for overall mortality, Table S4: Effect of surgical disparities (no treatment vs. local ablation/nephrectomy) on association between race/ethnicity and overall survival, Table S5; Association between neighborhood socioeconomic factors and surgical patterns in Non-Hispanic Blacks and Non-Hispanic Whites, Table S6; Association between neighborhood socioeconomic factors and overall mortality in Non-Hispanic Blacks and Non-Hispanic Whites.
