# Supplementary material for: Impacts of Neighborhood Characteristics and Surgical Treatment Disparities on Overall Mortality in Stage I Renal Cell Carcinoma Patients
Source: Int J Environ Res Public Health. 2022 Feb 12;19(4):2050. doi: 10.3390/ijerph19042050 (PMC8872003; doi:10.3390/ijerph19042050)
Supplement: Supplementary file 1 [file ijerph-19-02050-s001.zip › ijerph-1509509-supplementary.pdf]

Supplementary Materials

Impacts of Neighborhood Characteristics and Surgical Treatment Disparities on Overall Mortality in Stage I Renal Cell Carcinoma Patients

Alejandro Cruz, Faith Dickerson, Kathryn R. Pulling, Kyle Garcia, Francine C. Gachupin, Chiu-Hsieh Hsu, Juan Chipollini, Benjamin R. Lee, and Ken Batai

Table S1. Stage I RCC patient characteristics across Hispanic subgroups.

Characteristics of Patients, n (%)	Mexican/ Chicano (n=1,957)	Puerto Rican (n=535)	Cuban (n=432)	South or Central America (n=848)	Dominican (n=166)	*p*
Age, median (IQR)	58 (49-67)	60 (49-69)	64 (54-74)	58 (48-67)	62 (53-71)	<0.001
Gender						0.50
Male	1,135 (58.0)	308 (57.6)	269 (62.3)	486 (57.3)	98 (59.0)	
Female	822 (420)	227 (42.4)	163 (37.7)	362 (42.7)	68 (41.0)	
Grade, n (%)						0.26
1 & 2	1,171 (77.7)	305 (76.3)	244 (81.1)	511 (80.0)	73 (73.0)	
3 & 4	336 (22.3)	95 (23.8)	57 (18.9)	128 (20.0)	27 (27.0)	
Histologic Subtype						<0.001
Clear Cell	1,208 (84.4)	277 (70.3)	208 (69.1)	503 (79.0)	53 (49.5)	
Papillary	104 (7.3)	64 (16.2)	53 (17.6)	62 (9.7)	32 (29.9)	
Chromophobe	84 (5.9)	39 (9.9)	26 (8.6)	53 (8.3)	18 (16.8)	
Other	35 (2.4)	14 (3.6)	14 (4.7)	19 (3.0)	4 (3.7)	
Insurance Type						<0.001
Private	732 (37.4)	183 (34.2)	136 (31.5)	349 (41.2)	50 (30.1)	
Public	969 (49.5)	332 (62.1)	248 (57.4)	372 (43.9)	107 (64.5)	
Not insured	197 (10.1)	14 (2.6)	44 (10.2)	114 (13.4)	7 (4.2)	
Unknown	59 (3.0)	6 (1.1)	4 (0.9)	13 (1.5)	2 (1.2)	
Facility Type						<0.001
Community Cancer Program	184 (10.4)	36 (7.2)	6 (1.4)	56 (7.3)	30 (19.2)	
Comprehensive Community Cancer Program	736 (41.5)	123 (24.7)	85 (20.2)	168 (21.8)	30 (19.2)	
Academic/Research Program	780 (44.0)	296 (59.4)	114 (27.1)	421 (54.7)	84 (53.8)	
Integrated Network Cancer Program	72 (4.1)	43 (8.6)	216 (51.3)	125 (16.2)	12 (7.7)	
County-level Residence Pattern						<0.001
Metropolitan	1,791 (93.1)	506 (97.3)	422 (98.8)	824 (99.8)	156 (98.1)	
Urban	126 (6.5)	13 (2.5)	5 (1.2)	2 (0.2)	3 (1.9)	
Rural	7 (0.4)	1 (0.2)	0 (0.0)	0 (0.0)	0 (0.0)	
Median Income Quartiles						<0.001
<$38,000	438 (22.4)	164 (31.0)	135 (31.4)	134 (16.0)	69 (41.6)	
$38,000-$47,999	488 (25.0)	91 (17.2)	92 (21.4)	158 (18.8)	26 (21.7)	
$48,000-$62,999	597 (30.6)	150 (28.4)	120 (27.9)	237 (28.2)	36 (21.7)	
$63,000+	429 (22.0)	124 (23.4)	83 (19.3)	311 (37.0)	25 (15.1)	
% No High School Degree						<0.001
≥21%	1,050 (53.8)	215 (40.6)	224 (52.1)	332 (39.5)	104 (62.7)	
13.0-20.9%	409 (21.0)	154 (29.1)	108 (25.1)	199 (23.7)	29 (17.5)	
7.0-12.9%	327 (16.8)	111 (21.0)	60 (14.0)	184 (21.9)	24 (14.5)	
<7.0%	166 (8.5)	49 (9.3)	38 (8.8)	125 (14.9)	9 (5.4)	

Table S2. Logistic regression assessing undergoing radical nephrectomy vs. partial nephrectomy stratified by neighborhood characteristics.

Race/ethnicity	OR (95% C.I.)	*p*	OR (95% C.I.)	*p*	*p*-Interaction
Median Income	<47,999		≥$48,000		0.003
NHW	Reference		Reference		
AI/AN	1.38 (1.11-1.72)	0.004	1.11 (0.81-1.52)	0.51	
NHB	1.36 (1.29-1.43)	<0.001	1.44 (1.35-1.53)	<0.001	
Asian American	0.99 (0.88-1.22)	0.91	1.17 (1.06-1.29)	0.003	
HA	1.22 (1.15-1.29)	<0.001	1.06 (1.01-1.11)	0.03	
% No High School Degree	≥13.0%		<13.0%		0.35
NHW	Reference		Reference		
AI/AN	1.35 (1.07-1.70)	0.01	1.14 (0.85-1.52)	0.39	
NHB	1.38 (1.31-1.45)	<0.001	1.39 (1.30-1.48)	<0.001	
Asian American	1.16 (1.01-1.33)	0.04	1.16 (1.03-1.30)	0.02	
HA	1.18 (1.09-1.21)	<0.001	1.09 (1.03-1.15)	0.003	

Adjusting for age category, gender, RCC histologic subtype, facility type, insurance type, Charlson/Deyo Score, Year of Diagnosis, urban/rural residence, Great Circle Distance, and neighborhood characteristics (median income or high school education).

Table S3. Cox Regression analysis for overall mortality.

	Unadjusted		Adjusted model 1		Adjusted model 2		Adjusted model 3	
	HR (95%CI)	*p*	HR (95%CI)	*p*	HR (95%CI)	*p*	HR (95%CI)	*p*
Surgical Treatment								
Local Ablation or Nephrectomy	Reference				Reference			
No Treatment	5.76 (5.62-5.91)	<0.001			3.02 (2.80-3.26)	<0.001		
Nephrectomy Type								
Partial Nephrectomy	Reference						Reference	
Radical Nephrectomy	1.81 (1.77-1.86)	<0.001					1.52 (1.47-1.58)	<0.001
NHW vs. Racial/ethnic Minority Groups								
NHW	Reference		Reference		Reference		Reference	
AI/AN	0.99 (0.84-1.16)	0.86	0.99 (0.79-1.26)	0.99	1.01 (0.80-1.27)	0.96	0.90 (0.69-1.18)	0.45
NHB	1.14 (1.10-1.17)	<0.001	1.11 (1.06-1.17)	<0.001	1.11 (1.05-1.16)	<0.001	1.08 (1.02-1.14)	0.007
Asian American	0.66 (0.59-0.73)	<0.001	0.73 (0.63-0.84)	<0.001	0.72 (0.63-0.83)	<0.001	0.69 (0.59-0.81)	<0.001
HA	0.83 (0.81-0.86)	<0.001	0.87 (0.83-0.91)	<0.001	0.87 (0.83-0.92)	<0.001	0.85 (0.80-0.90)	<0.001
NHWs vs. HA Subgroups								
NHWs	Reference		Reference		Reference		Reference	
Mexican/Chicano	0.83 (0.73-0.93)	0.002	0.95 (0.80-1.12)	0.51	0.96 (0.81-1.13)	0.62	0.91 (0.75-1.09)	0.30
Puerto Rican	0.67 (0.52-0.87)	0.002	0.55 (0.37-0.82)	0.003	0.56 (0.37-0.83)	0.004	0.58 (0.37-0.92)	0.02
Cuban	0.87 (0.69-1.10)	0.24	0.74 (0.53-1.04)	0.09	0.72 (0.52-1.02)	0.06	0.79 (0.54-1.15)	0.22
South or Central American	0.53 (0.43-0.37)	<0.001	0.71 (0.53-0.97)	0.03	0.71 (0.52-0.97)	0.03	0.75 (0.53-1.06)	0.10
Dominican	0.73 (0.47-1.15)	0.18	0.76 (0.40-1.47)	0.42	0.73 (0.38-1.41)	0.35	0.69 (0.33-1.46)	0.33

Model 1: model includes age category, gender, RCC histologic subtype (NOS not included), grade (1 and 2 vs. 3 and 4), facility type, insurance type, great circle distance, neighborhood characteristics (median income and % high school graduation), Charlson/Deyo Score, and Year of Diagnosis.
Model 2: Model 1 + no surgical treatment vs. treatment; no surgery compared to surgical treatment (HR 3.02, 95% CI: 2.80-3.26)
Model 3: Model 1 + radical vs. partial nephrectomy; Radical compared to partial nephrectomy (HR 1.52, 95% CI: 1.47-1.58)

Table S4. Effect of surgical disparities (no treatment vs. local ablation/nephrectomy) on association between race/ethnicity and overall survival.

**Race/ethnicity**	**No Treatment**		**Treatment**		
	**HR (95%CI)**	***p***	**HR (95%CI)**	***p***	$p_{\text{Interaction}}$
NHW	Reference		Reference		0.24
NHB	1.01 (0.79-1.28)	0.96	1.11 (1.06-1.17)	<0.001	
HA	0.88 (0.68-1.15)	0.37	0.87 (0.83-0.91)	<0.001	

Regression model includes age category, gender, RCC histologic subtype, grade (1 and 2 vs. 3 and 4), facility type, insurance type, great circle distance, neighborhood characteristics (median income and % high school graduation), Charlson/Deyo Score, and Year of Diagnosis.

Table S5. Association between neighborhood socioeconomic factors and surgical patterns in Non-Hispanic Blacks and Non-Hispanic Whites.

	**No treatment**		**Radical vs. Partial**	
	**OR (95% CI)**	***p***	**OR (95% CI)**	***p***
Non-Hispanic Blacks				
% No High School Degree				
≥21%	Reference		Reference	
1.30-20.9%	1.10 (0.91-1.34)	0.32	1.12 (1.03-1.22)	0.01
7.0-12.9%	1.23 (0.95-1.60)	0.12	0.94 (0.84-1.06)	0.35
<7.0%	1.27 (0.87-1.86)	0.22	0.97 (0.83-1.14)	0.70
Median Income				
<$38,000	Reference		Reference	
$38,000-$47,000	0.94 (0.76-1.16)	0.57	0.95 (0.86-1.05)	0.29
$48,000-$62,000	0.81 (0.63-1.03)	0.08	1.03 (0.92-1.15)	0.62
$63,000+	0.56 (0.41-0.77)	<0.001	0.96 (0.94-1.10)	0.60
Non-Hispanic Whites				
% No High School Degree				
≥21%	Reference		Reference	
1.30-20.9%	0.97 (0.85-1.10)	0.60	0.96 (0.91-1.01)	0.08
7.0-12.9%	1.03 (0.90-1.18)	0.66	0.91 (0.86-0.96)	<0.001
<7.0%	0.94 (0.81-1.10)	0.43	0.88 (0.82-0.93)	<0.001
Median Income				
<$38,000	Reference		Reference	
$38,000-$47,000	0.92 (0.82-1.04)	0.20	0.96 (0.91-1.01)	0.12
$48,000-$62,000	0.82 (0.72-0.94)	0.004	0.95 (0.90-1.01)	0.08
$63,000+	0.80 (0.69-0.93)	0.003	0.89 (0.84-0.95)	<0.001

NHBs – no treatment vs. treatment - age category, sex, facility type, insurance type, year of surgery, RCC histologic subtype, and comorbidity.

NHBs – radical vs. partial – age category, sex, facility type, insurance type, year of surgery, RCC histologic subtype, comorbidity, and urban/rural.
NHWs – no treatment vs. treatment – age category, sex, facility type, insurance type, year of surgery, RCC histologic subtype, comorbidity, and urban/rural.
NHWs – radical vs. partial – age category, sex, facility type, insurance type, year of surgery, RCC histologic subtype, comorbidity, urban/rural, and great distance.

Table S6. Association between neighborhood socioeconomic factors and overall mortality in Non-Hispanic Blacks and Non-Hispanic Whites.

	**Model 1**		**Model 2**	
	**HR (95% CI)**	***p***	**HR (95% CI)**	***p***
Non-Hispanic Blacks				
% No High School Degree				
≥21%	Reference		Reference	
1.30-20.9%	0.99 (0.90-1.11)	0.97	0.99 (0.88-1.12)	0.94
7.0-12.9%	1.04 (0.90-1.21)	0.56	0.99 (0.84-1.17)	0.91
<7.0%	0.93 (0.75-1.15)	0.49	0.93 (0.72-1.19)	0.57
Median Income				
<$38,000	Reference		Reference	
$38,000-$47,000	0.93 (0.83-1.04)	0.18	0.87 (0.76-0.99)	0.03
$48,000-$62,000	0.78 (0.68-0.89)	<0.001	0.76 (0.65-0.89)	<0.001
$63,000+	0.75 (0.63-0.89)	0.001	0.74 (0.61-0.91)	0.004
Non-Hispanic Whites				
% No High School Degree				
≥21%	Reference		Reference	
1.30-20.9%	0.94 (0.89-0.99)	0.04	0.94 (0.88-1.01)	0.07
7.0-12.9%	0.90 (0.84-0.95)	<0.001	0.90 (0.84-0.97)	0.003
<7.0%	0.82 (0.76-0.88)	<0.001	0.83 (0.76-0.90)	<0.001
Median Income				
<$38,000	Reference		Reference	
$38,000-$47,000	0.96 (0.91-1.02)	0.15	0.95 (0.89-1.02)	0.14
$48,000-$62,000	0.90 (0.85-0.95)	<0.001	0.89 (0.83-0.95)	<0.001
$63,000+	0.79 (0.74-0.85)	<0.001	0.79 (0.73-0.85)	<0.001

NHBs - Age cat, sex, facility type, insurance type, comorbidity, year of diagnosis, RCC histologic subtype, and no treatment vs. treatment (Model 1) or radical/partial nephrectomy (Model 2).
NHWs - Age cat, sex, facility type, insurance type, comorbidity, year of diagnosis, RCC histologic subtype, distance, and no treatment vs. treatment (Model 1) or radical/partial nephrectomy (Model 2).
